# Differentiation of Glial Cells From hiPSCs: Potential Applications in Neurological Diseases and Cell Replacement Therapy

**DOI:** 10.3389/fncel.2018.00239

**Published:** 2018-08-08

**Authors:** Wei Zheng, Qian Li, Chao Zhao, Yuwei Da, Hong-Liang Zhang, Zhiguo Chen

**Affiliations:** ^1^Cell Therapy Center, Xuanwu Hospital, Capital Medical University, Beijing, China; ^2^Key Laboratory of Neurodegeneration, Ministry of Education, Beijing, China; ^3^Department of Clinical Neurosciences, Wellcome Trust-Medical Research Council Stem Cell Institute, University of Cambridge, Cambridge, United Kingdom; ^4^Department of Neurology, Xuanwu Hospital, Capital Medical University, Beijing, China; ^5^Department of Life Sciences, National Natural Science Foundation of China, Beijing, China; ^6^Center of Neural Injury and Repair, Beijing Institute for Brain Disorders, Beijing, China

**Keywords:** astrocytes, oligodendrocytes, microglia, hiPSC, differentiation

## Abstract

Glial cells are the most abundant cell type in the central nervous system (CNS) and play essential roles in maintaining brain homeostasis, forming myelin, and providing support and protection for neurons, etc. Over the past decade, significant progress has been made in the reprogramming field. Given the limited accessibility of human glial cells, *in vitro* differentiation of human induced pluripotent stem cells (hiPSCs) into glia may provide not only a valuable research tool for a better understanding of the functions of glia in the CNS but also a potential cellular source for clinical therapeutic purposes. In this review, we will summarize up-to-date novel strategies for the committed differentiation into the three major glial cell types, i.e., astrocyte, oligodendrocyte, and microglia, from hiPSCs, focusing on the non-neuronal cell effects on the pathology of some representative neurological diseases. Furthermore, the application of hiPSC-derived glial cells in neurological disease modeling will be discussed, so as to gain further insights into the development of new therapeutic targets for treatment of neurological disorders.

## Introduction

Glial cells are the most abundant cell type in the CNS with a remarkable role both in structure maintenance and functioning. Glial cells were initially suggested as so-called “nervenkitt” or nerve-cement, which was first promulgated as a concept in a lecture delivered by Rudolf Virchow more than 160 years ago. Virchow defined the term “neuroglia” (synonym of glial cells) which originated from the Greek word “glía” meaning “glue” in English (Kettenmann and Verkhratsky, 2008). An increasing volume of literature has revealed that glial cells are involved in almost every aspect of neural activity and play critical roles in CNS functions, development, injury, and diseases (Zhang, 2001; Barres, 2008; Jäkel and Dimou, 2017).

Glial cells were traditionally divided into three categories: astrocytes, oligodendrocytes (OLs), and microglia. NG2-glia, also known as polydendrocytes, have been identified as a new class of glial cells that express the NG2 proteoglycan, and been recognized as oligodendrocyte progenitor cells (OPCs) (Paukert and Bergles, 2006; Nishiyama, 2007; Oberheim et al., 2012). In addition to these major classes of glial cells, some specialized glial cells in distinct areas of brain have also been identified, such as Müller glia in retina and Bergmann glia in cerebellum (Kettenmann and Verkhratsky, 2008).

In this review, we will describe recent advances addressing the roles of the three major neuroglia with respect to their participation in brain functions, development, and diseases. Research of human neuroglia development and function has been hindered by a constellation of challenges, for instance, the difficulty in the tracing of endogenous glial cells, including live or post-mortem tissues. In most circumstances, isolation of one type of neuroglia from post-mortem tissues is difficult; for example, the first human astrocytes isolated from post-mortem tissue (Ennas et al., 1992) were unexpectedly contaminated with other cells including microglia. A major hurdle is the lack of purification methods to separate neuroglia (Zhang et al., 2016). Given the limited accessibility to human brain tissues, rodent glial cells have frequently served as a substitute to deduce the functions of human glial cells. However, recent studies demonstrate that rodent neuroglia are insufficient to model human neuroglia-related diseases/disorders, due to the large differences in the distribution of neuroglia types, neuroanatomy, and neurogenesis between rodents and humans (Oberheim et al., 2009). Moreover, many current methods applied for isolation may alter the phenotypes of live glial cells as well (Muffat et al., 2016). In light of these challenges, developing *in vitro* human glial cell models may be of great significance. iPSCs have emerged as a valuable tool for modeling diseases, studying the development of human CNS, and exploring treatment modalities for CNS disorders (Li and Izpisua Belmonte, 2016; Hamazaki et al., 2017; Shi et al., 2017). Yamanaka and colleagues were the first to achieve the reprogramming of human fibroblasts into iPSCs by using defined transcription factors (Takahashi et al., 2007). The great self-renewal and wide differentiation capacities of iPSCs make it possible to obtain large numbers of variable cell types in a cell-type-specific manner. Meanwhile, iPSCs can be generated from patients' blood cells, skin fibroblasts, and other somatic cell sources, providing unlimited and valuable disease-related cells in a personalized manner. In this regard, generating glial cells from iPSCs has opened up a new area for investigating the critical roles of glial cells in CNS development, function, and disease at a cellular level (Chandrasekaran et al., 2016; Gonzalez et al., 2017). Glial cells are involved in many neurological diseases, such as amyotrophic lateral sclerosis (ALS), spinal cord injury (SCI), Alzheimer's disease (AD), and multiple sclerosis (MS). hiPSC-derived glial cells may provide new and ethically acceptable cell sources for glial cell replacement therapy.

**Figure 1:**
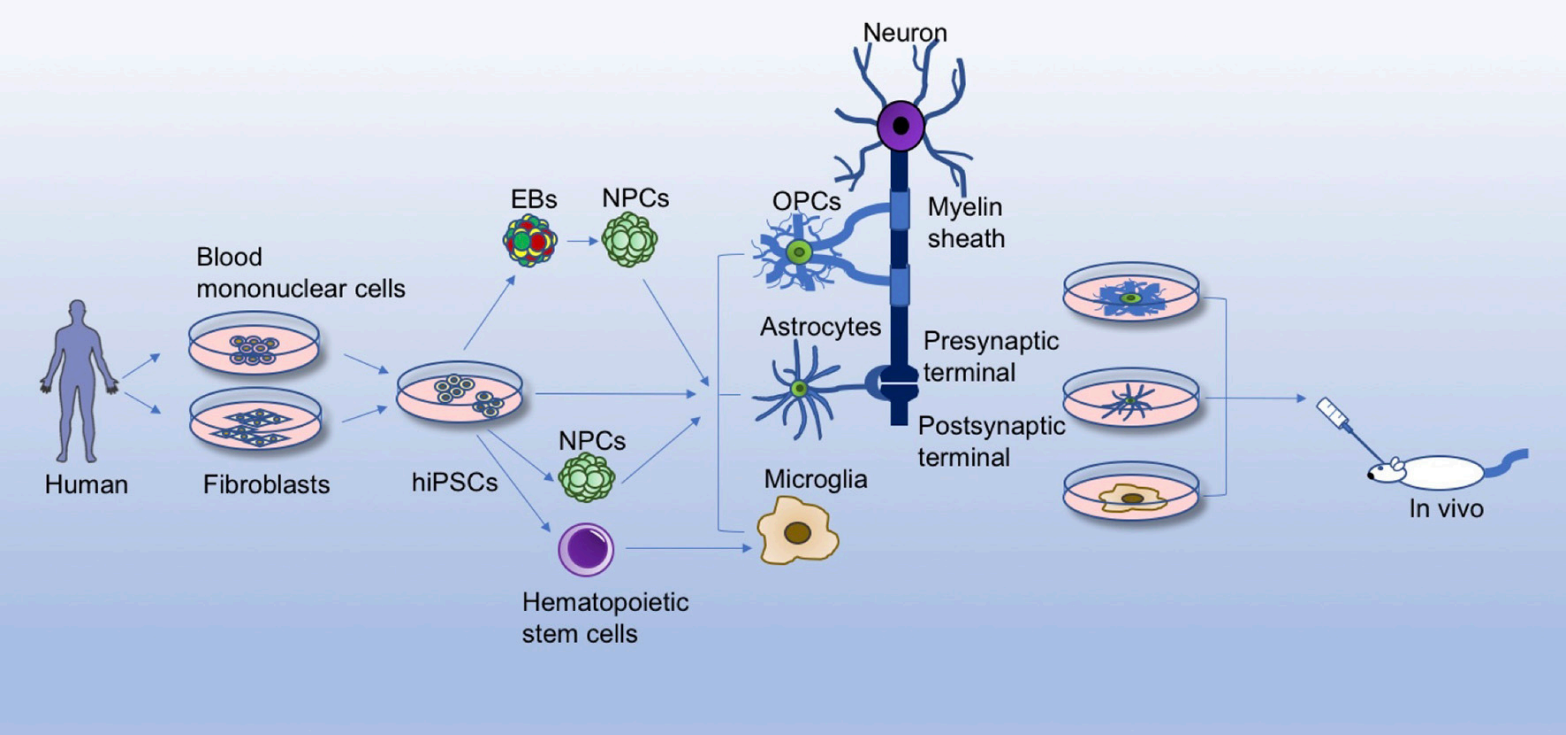
Differentiation and application of hiPSC-derived glial cells. Human iPSCs (hiPSCs) reprogrammed from healthy person/patient-derived somatic cells can give rise to glial cells through different differentiation methods, for instance, by directly differentiating hiPSCs into glial cells, or by first inducing hiPSCs into embryoid bodies (EBs), neural progenitor cells (NPCs), or hematopoietic stem cells. hiPSC-derived glial cells offer a platform for studying the physiology of glia, disease mechanisms, and glia/neuron interactions in a culture dish. Accumulating evidence has revealed crucial roles of glial cells in the brain, such as rapid and saltatory conduction, motor skill learning, energy/trophic support, synapse formation, innate immune system function, etc. Meanwhile, hiPSC-derived glial cells can be transplanted into animal models (amytrophic lateral sclerosis, spinal cord injury, Alzhemier's disease, multiple sclerosis, etc.) to evaluate the safety and efficacy for treatment of these diseases.

In this review, we first briefly discuss the current knowledge of glial cells in brain functions and developments, and then summarize advances in generating the three major neuroglia cell types (astrocytes, OLs, and microglia) from hiPSCs, with emphasis on the non-neuronal cell effects on the pathology of some representative neurological diseases, so as to gain further insights into the development of new therapeutic targets for neurological disorders (Figure 1).

## Classification and origin of glial cells

Based on the current knowledge, there are mainly three types of glial cells: astrocytes, OLs, and microglia, differing in their origins, molecular markers, physiological functions and subtypes (summarized in Table 1). Astrocytes, as the most numerous type of glial cells, comprise a heterogeneous group of cell subtypes that have a crucial role in brain function and development. Based on our current understanding, astrocytes differ in gene expression, morphology, physiology, pathology, and metabolism amongst cortical regions (Doyle et al., 2008; Zhang and Barres, 2010; Khakh and Sofroniew, 2015). Astrocytes consist of at least four distinct subtypes of glial fibrillary acidic protein (GFAP)-positive cells in human brain, while two subtypes in rodents, indicating a large species difference among mammals.

**Table 1:** Subtypes, origins, molecular markers, and physiological functions of glial cells.

**Subtypes**	**Origins**	**Molecular markers**	**Functions**	**References**
Astrocytes (including protoplasmic astrocytes, interlaminar astrocytes, polarized astrocytes, and fibrous astrocytes)	Embryonic germ layer (also known as neuroectoderm); in details, two sources were reported about the origin of neurons: radial glial cells (RGCs) within the ventricular zone and intermediate progenitors in the subventricular zone.	•Glial fibrillary acid protein (GFAP) •S100β •Glutamate transporter 1 (GLT1) •Nuclear factor 1A •Vimentin •Aquaporin 4 •GLAST 1 •ALDH1	•Regulation of extracellular neurotransmitter levels (such as glutamate-glutamine cycle), water balance, excitotoxicity; •Control blood flow; •Supply energy to neurons; •Maintenance of the blood-brain barrier; •In synapse formation and synapse elimination that remodels neural circuits; •Participate in advanced brain functions.	Oberheim et al., 2006 Vasile et al., 2017 Allen and Barres, 2009 Ge et al., 2012 Coulter and Eid, 2012 MacVicar and Newman, 2015 Clarke and Barres, 2013 Krencik et al., 2011 Cao et al., 2013
Oligodendrocytes	Embryonic germ layer (also known as neuroectoderm); OPC generates from two origins, both of which have three waves: (1) Spinal cord-ventral region, dorsal neural tube, the specific zone of the third wave is unclear yet. (2) Forebrain-the medial ganglionic eminence and the anterior entopeduncular area, ventral ventricular zone of the telencephalon, cortex.	•Olig2 •NG2 •A2B5 •PDGFRα •O4 •MBP	•Myelination; •Rapid and saltatory conduction; •Energy/trophic metabolism support; •Maintenance of axonal structural integrity and “non-inflammatory” environment; •Motor skills learning; •Rapid response to demyelination injuries.	Cai et al., 2005 Fogarty et al., 2005 Rowitch and Kriegstein, 2010 Kessaris et al., 2006 Goldman and Kuypers, 2015 Hart et al., 1989 Waxman, 1977 Philips and Rothstein, 2017 Crawford et al., 2014 Kondo and Raff, 2000
Microglia (presented with dynamic diverse phenotypes, ranging from classically pro-inflammatory M1 phenotypes to alternatively anti-inflammatory M2 phenotypes)	Myeloid origin and derived from hematopoietic stem cells in the yolk sac.	•IBA1 •CD11b •CD45 •CD68 •TMEM119 •P2RY12	•Form the frontline defense of the CNS immune system; •Participate in synaptic pruning and synaptic plasticity; •Promote neuronal survival during development; •Through phagocytosis to eliminate resultant cellular debris in the cleanup system.	Tang and Le, 2016 Greter and Merad, 2013 Muffat et al., 2016 Glass et al., 2010 Wu et al., 2015 Colonna and Butovsky, 2017 Takahashi et al., 2005

According to the literature, the four subtypes of human astrocytes include: protoplasmic astrocytes found in the gray matter (layers 2–6 of cortex), interlaminar astrocytes (layer 1 of cortex), polarized astrocytes (layer 5–6 of cortex), fibrous astrocytes that populate the white matter (Oberheim et al., 2006; Vasile et al., 2017). Nevertheless, there are only protoplasmic and fibrous astrocytes identified in rodents (Oberheim et al., 2006; Vasile et al., 2017). In general, the processes of protoplasmic astrocytes ensheath synapses as well as blood vessels, while fibrillary (or fibrous) astrocytes ensheath blood vessels and contact with nodes of Ranvier (Barres, 2008).

OLs are a type of glial cells which distribute throughout the CNS, and they are generated from OPCs. OPCs were first isolated from rat optic nerves and defined as bi-potential OL-type-2 astrocytes, which are positive for A2B5, and can *in vitro* differentiate into either OLs or fibrous astrocytes in different culture conditions (Raff et al., 1983; Ffrench-Constant and Raff, 1986). OPCs, accounting for 3% of all cells in adult human brain, can be found in both the gray and white matter, but a larger percentage in the latter (Dawson, 2003; Crawford et al., 2014).

Microglia, the resident macrophages in the brain and spinal cord, form the innate immune system via active surveillance through phagocytosing debris and contributing to inflammation in response to various toxins, pathogens and injury (Tremblay et al., 2011; Nayak et al., 2014). As the main resident immune cells in the CNS, microglia activation serves as the major component of neuro-inflammation whenever pathogens invade, forming the frontline defense of the CNS immune system (Glass et al., 2010). When the resting microglia detect any signal of injury or tissue damage, microglia shift toward an activated state, termed polarization, and initiate innate immune responses (Durafourt et al., 2012; Prinz and Priller, 2014). Microglia presented with dynamic diverse phenotypes depending on the actions of modulators and signals detected. Microglia activation ranges from classically activated pro-inflammatory M1 phenotypes to alternatively activated anti-inflammatory M2 phenotypes (Tang and Le, 2016). Classically activated M1 phenotype microglia can be stimulated with lipopolysaccharide (LPS) in combination with interferon (IFN)-γ. And alternatively activated M2 phenotype microglia are normally driven by interleukin (IL)-4/IL-13 and IL-10/transforming growth factor (TGF)-β (Hu et al., 2012; Cunningham, 2013). In human brain, M1 microglia firstly take action to respond to pathogens and release pro-inflammatory factors, such as IL-6, IL-1β, IL-12, tumor necrosis factor (TNF)-α, and chemokine C-C motif ligand 2 (CCL2), which may result in neurotoxicity. In contrast, M2 microglia act as the major cells to antagonize inflammatory responses by releasing anti-inflammatory factors, such as IL-10 and TGF-β, promoting immunosuppression and neuronal protection (Tang and Le, 2016). However, balance of these two phenotypes could shift at different stages following injury and in neurodegenerative diseases, which will be discussed in the following part.

Gliogenesis are generally considered to occur following neurogenesis during human brain development. Astrocytes and OLs originate from the embryonic germ layer, also known as neuroectoderm, consistent with the origin of neurons (Allen and Barres, 2009). Furthermore, two sources were reported with respect to the origin of astrocytes: radial glial cells (RGCs) within the ventricular zone and intermediate progenitors in the subventricular zone (Ge et al., 2012). First, RGCs generate new neurons and intermediate progenitors within ventricular zone, and then the intermediate progenitors generate neurons or glial cells. RGCs can switch to gliogenesis to produce astrocytes, after being activated by factors such as bone morphogenetic protein (BMP)-4 and IL-6 (Chandrasekaran et al., 2016). As to the origin of OLs, OPC genesis first takes place in the spinal cord (Goldman and Kuypers, 2015). OPCs start in the motor neuron progenitor domain of the ventral region, a process dependent on sonic hedgehog (SHH). This generation phase requires the expression of oligodendrocyte transcription factor (Olig) 2, the basic helix-loop-helix transcription factor, which is regulated by Nkx6 (Takebayashi et al., 2002). In mouse spinal cord, this ventrally derived subpopulation makes up ~80% of all OPCs (Tripathi et al., 2011). The second wave of OPC production is derived from dorsal neural tube, relying on fibroblast growth factor (FGF) but independent of SHH signaling or Nkx6 (Cai et al., 2005; Fogarty et al., 2005). Finally, the third wave of OPC origination occur at birth (Rowitch and Kriegstein, 2010). Within the forebrain, production of OPCs is generally similar to that in spinal cord. In the beginning, OPCs are generated from Nkx2.1-expressing precursors of the medial ganglionic eminence and the anterior entopeduncular area; and then these cells migrate gradually to the entire telencephalon and the cortex (Kessaris et al., 2006). The subsequent wave appears in lateral and/or caudal ganglionic eminences of ventral ventricular zone of the telencephalon which expresses Gsh2 (Kessaris et al., 2006). During this phase, the downregulation of Gsh2 (also known as Gsx2) is essential for OPC generation, since it can directly repress the expression of platelet-derived growth factor receptor alpha (PDGFRα), a marker of OPCs (Hart et al., 1989; Chapman et al., 2013; Goldman and Kuypers, 2015). The final stage of OPC production arises from cortex around birth which is under the transcriptional control of Emx1. In comparison with astrocytes and OLs, the origin and the lineage of microglia have been a subject of debate after their discovery. In fact, the viewpoint that microglia are of myeloid origin and derived from hematopoietic stem cells in the yolk sac is favored by a majority (Hickey and Kimura, 1988; Simard and Rivest, 2004; Greter and Merad, 2013). As the resident macrophages of CNS, microglia display unique mesodermal ontogeny differing from other adult tissue monocyte-derived macrophages (Muffat et al., 2016). Moreover, more definitive evidence suggests that human microglia originate from primitive myeloid precursors emanating from the yolk sac and arise before embryonic day 8 in the developmental period, as demonstrated by fate-mapping analysis (Ginhoux et al., 2010; Prinz and Priller, 2014).

## The physiological role of glial cells

Glial cells are indispensable for brain homeostasis, development, function, and are involved in a variety of neurological diseases. Here we will discuss the functions of glial cells under physiological conditions, including balancing ion concentration, supplying energy, buffering pH, remodeling synapses and recycling neurotransmitters, supporting neurons and forming the innate immune system.

In comparison with rodents, astrocytes in human cortex exhibit greater heterogeneity and diversity, and display differences in morphology, physiology, gene expression profile, and electrophysiological properties (Zhang and Barres, 2010; Khakh and Sofroniew, 2015). Astrocytes maintain brain homeostasis by multiple dynamic equilibrium adjustments, including regulation of extracellular neurotransmitter levels, water balance, excitotoxicity, and control of blood flow (Coulter and Eid, 2012). Among the neurotransmitters, 80–90% of extracellular glutamate is uptaken by astrocytes, transformed to glutamine by glutamine synthetase, and finally converted back to glutamate after being uptaken into GABAergic and glutamatergic neurons, known as glutamate-glutamine cycle (Coulter and Eid, 2012; Parpura and Verkhratsky, 2012). Besides, together with endothelial cells as well as pericytes, astrocytes form the blood-brain barrier, with their endfoot processes enveloping brain blood vessels (MacVicar and Newman, 2015). Additionally, astrocytes supply energy to neurons through ferrying glucose and oxygen from blood and converting them to lactate, known as one of the oldest function of astrocytes (Allen and Barres, 2009). Astrocytes are also actively involved in the formation of synapses via direct contact with neurons and indirectly regulate synapse formation through cytokine factors (Clarke and Barres, 2013). Moreover, astrocytes play essential roles in synapse elimination that remodels neural circuits during CNS development (Chung et al., 2013). With regard to the electrophysiological functions, astrocytes are not electrically excitable and therefore do not generate action potentials. Yet the resting membrane potential of human astrocytes are sustained at −63.9 to −70 mV, while ~-80 mV in rodent astrocytes (Oberheim et al., 2012; Vasile et al., 2017). Especially, astrocytes are demonstrated to actively participate in advanced brain functions, including coordinating motor functions (Saab et al., 2012), modulating depressive-like behaviors (Cao et al., 2013), regulating memory (Orr et al., 2015), modulating aging (Yin et al., 2017), as well as controlling circadian behavioral rhythms and sleep-wake cycles (Ding et al., 2016; Brancaccio et al., 2017). However, due to their diversity, future work is required for a full characterization of astrocytes.

In the CNS, OLs play a crucial role in supporting neurons. OLs form myelin, a multi-lamellar lipid structure with high-resistance, to wrap neuronal axons, thereby ensuring rapid and saltatory conduction. Demyelinated fibers could lead to decreased conduction velocity or conduction failure (Waxman, 1977). Between myelinated segments formed by OLs along an axon or on different axons are unmyelinated portions named nodes of Ranvier. In addition to their function in saltatory conduction, more evidence suggests that OLs can also provide energy support to neurons by being involved in pyruvate cycling and ATP synthesis, and NG2-OPCs provide support by being involved in glutamate signaling and homeostasis in the CNS (Dawson, 2003; Fünfschilling et al., 2012; Lee et al., 2012; Saab et al., 2013; Philips and Rothstein, 2017). Myelinating OLs are also critical for the structural integrity of axons and maintain a “non-inflammatory” environment (Hirrlinger et al., 2002; Kassmann et al., 2007). Myelination persists in human brain from birth to adulthood. By testing maximal finger tapping speed which depends on the myelination process and the integrity of myelin, it was shown that the maximum motor speed appears in middle-aged men and declines in older ones (Bartzokis et al., 2010). OPCs are the major cell source of myelination. Within the adults CNS, OPCs could be activated from a quiescent state by demyelination injuries and take rapid responses (Crawford et al., 2014). OPCs would proliferate, migrate, and differentiate into mature myelinating OLs and ultimately remyelination is achieved. Notably, remyelination efficiency declines with age, as characterized by the decreasing rate of cell division, the delay in OPC recruitment and differentiation, and the decline of myelin content and integrity in brain (Sim et al., 2002; Bartzokis et al., 2010; Richardson et al., 2011; Young et al., 2013). Another notable phenomenon is that the myelin internodes become shorter but the number of the nodes increases with age (Young et al., 2013). The cause of this phenomenon is unclear, whereas it was proposed that the change may be related to the speed of conduction. Furthermore, myelination is associated with motor skill learning. For example, OPC proliferation and OL production have been shown in the corpus callosum of mice, which is triggered by a novel experience running on complex wheels with irregularly spaced rungs (McKenzie et al., 2014; Xiao et al., 2016). The proliferation of OPCs seems to be influenced by the electrical activity of axons in close proximity (Barres and Raff, 1993). OPCs could form direct synapses with neurons in both gray and white matters and they have the potential to differentiate into astrocytes or neurons under specific conditions (Raff et al., 1983; Ffrench-Constant and Raff, 1986; Kondo and Raff, 2000). OPCs can also transform into cancer cells caused by expression or deletion of some specific genes (Bergles and Richardson, 2016).

Microglia facilitate the wiring of neural networks via migration into the CNS as the first glial cells (Colonna and Butovsky, 2017). During prenatal development, microglia as guidepost cells play significant roles at the crossroads of axonal fiber pathways and neural migratory routes in guiding neurons and axons to form neural circuits (Squarzoni et al., 2015). Furthermore, emerging evidence indicates that microglia take active part in the developmental synaptic pruning and synaptic plasticity (Wu et al., 2015). Deficiency of a transmembrane polypeptide DAP12, restrictedly expressed in microglia, impaired synaptic function through reducing the expression of brain-derived neurotrophic factor-tyrosine kinase receptor B (Roumier et al., 2004). Microglial behavior could increase phagocytosis of synaptic elements and interact with subsets of structurally dynamic and transient synapses (Wake et al., 2009; Tremblay et al., 2010). Under two-photon *in vivo* imaging and electron microscopy, processes of microglia actively contact with dendritic spines, highlighting the role of microglia in synaptic activity and plasticity (Tremblay et al., 2010). Some trophic factors and synaptogenic signals derived from microglia are significant for synaptic pruning. Moreover, microglia can release some trophic factors to promote the survival of surrounding neurons. For example, insulin-like growth factor-1 derived from microglia supports the survival of layer 5 cortical neurons (Ueno et al., 2013). Besides, microglia secrete some growth factors, include FGF, and other neurotrophic factors like glial cell-derived neurotrophic factor, brain-derived neurotrophic factor and nerve growth factors, which are essential facilitators of neuronal growth and health (Colonna and Butovsky, 2017). Nevertheless, microglia not only promote neuronal survival during development, but are also in part responsible for inducing neuronal death. In fact, approximately half of the immature or faulty neurons caused by improper migration or differentiation assume programmed cell death and require elimination (Nayak et al., 2014). As expected, microglia play an active role in the cleanup system through phagocytosis to eliminate resultant cellular debris (Takahashi et al., 2005; Hristova et al., 2010); after status epilepticus in which situation the number of newborn cells increases rapidly, for example, microglia are rapidly activated and eliminate some newborn cells in the subgranular zone by phagocytosis (Luo et al., 2016). Taken together, microglia perform indispensable roles and contribute to the microenvironment homeostasis and the dynamic change of neural circuits via secreting various neurotoxic or neuroprotective factors involved in the process of induced neuronal death/clean up or neurogenesis.

Appropriate interaction between neurons and glia is crucial for the maintenance of the nervous system homeostasis. A commonsense notion is that glial cells constitute about 90% of all cells in human brain and glia/neuron ratio increases with brain size across different species (Ullian et al., 2001; Nishiyama et al., 2005). Yet this notion has been challenged by Herculano-Houzel et al. who proposed that the glia/neuron ratio does not increase with brain size but with decreasing neuronal density, and human brain consists of roughly 50% neuronal and 50% non-neuronal cells (Herculano-Houzel, 2014).

In most studies, mouse- and rat-derived glial cells have been employed as the primary subject to study the biology and pathology of human glia. In spite of some similarities, it is also noteworthy to stress the fundamental differences in human vs. rodent glial cells, which may have accounted for the numerous failures of drug discovery in translating rodent studies to humans. (i) Different subtypes of astrocytes exist between human and rodent astrocytes. Only protoplasmic and fibrous astrocytes have been identified in rodents, whereas two additional subtypes, interlaminar and polarized astrocytes, have been identified in human (Oberheim et al., 2009). (ii) Human astrocytes cover larger territories than do rodent astrocytes. Oberheim et al. first found that human astrocytes are significantly larger in size than rodent astrocytes *in vivo*. Then to find out whether intrinsic qualities or non-cell-autonomous pathways contribute to the larger size, Zhang et al. purified astrocytes by immunopanning and cultured them *in vitro*, and showed that the length of human astrocyte processes are almost twice of that of rodent cells *in vitro* (Zhang et al., 2016). (iii) Human astrocytes have almost twice the number of branches as many as rodent astrocytes, which are 8.5 ± 1.1 and 4.5 ± 0.5, respectively (Zhang et al., 2016). (iv) Human astrocytes are 2.6-fold larger in diameter than rodent astrocytes. (v) The ratio of glial cells to neurons is much larger in human vs. in rodent, which are ~1.65:1 in human and ~0.3:1 in rodents, respectively (Nedergaard et al., 2003; Sherwood et al., 2006). (vi) As rodent astrocytes do, human astrocytes respond to glutamate and ATP through calcium waves, but with a faster speed for propagating calcium signals (Zhang et al., 2016). Albeit the above, it remains an intriguing question whether such a difference leads to distinction of cognitive abilities between human and rodent animals. Also, given the high frequency of drug discovery failures in translation of rodent data to humans, the difference of glia between species should be taken into consideration, particularly in translational studies. To overcome these obstacles, generating glial cells from hiPSCs may provide a solution.

## Current appoaches for glial differentiation from hiPSCs

### Astrocytes

Research of glial cells related to neurological diseases has been hindered by some challenges as listed below: limited accessibility of neural tissues, difficulties in culturing glia *in vitro* (current isolation method may change the phenotypes), and the large difference between rodent models and human diseases due to the distinctions in glial cell morphology, distribution, and function between species (Oberheim et al., 2006, 2009). hiPSCs are at the forefront of research in neurological diseases and are recognized as a source for generating autologous donor cells for disease modeling, drug discovery, and replacement therapy (Kikuchi et al., 2017). Improved differentiation of glial cells from hiPSCs can generate a large number of glial cells in an iterative and controlled manner.

**Figure 2:**
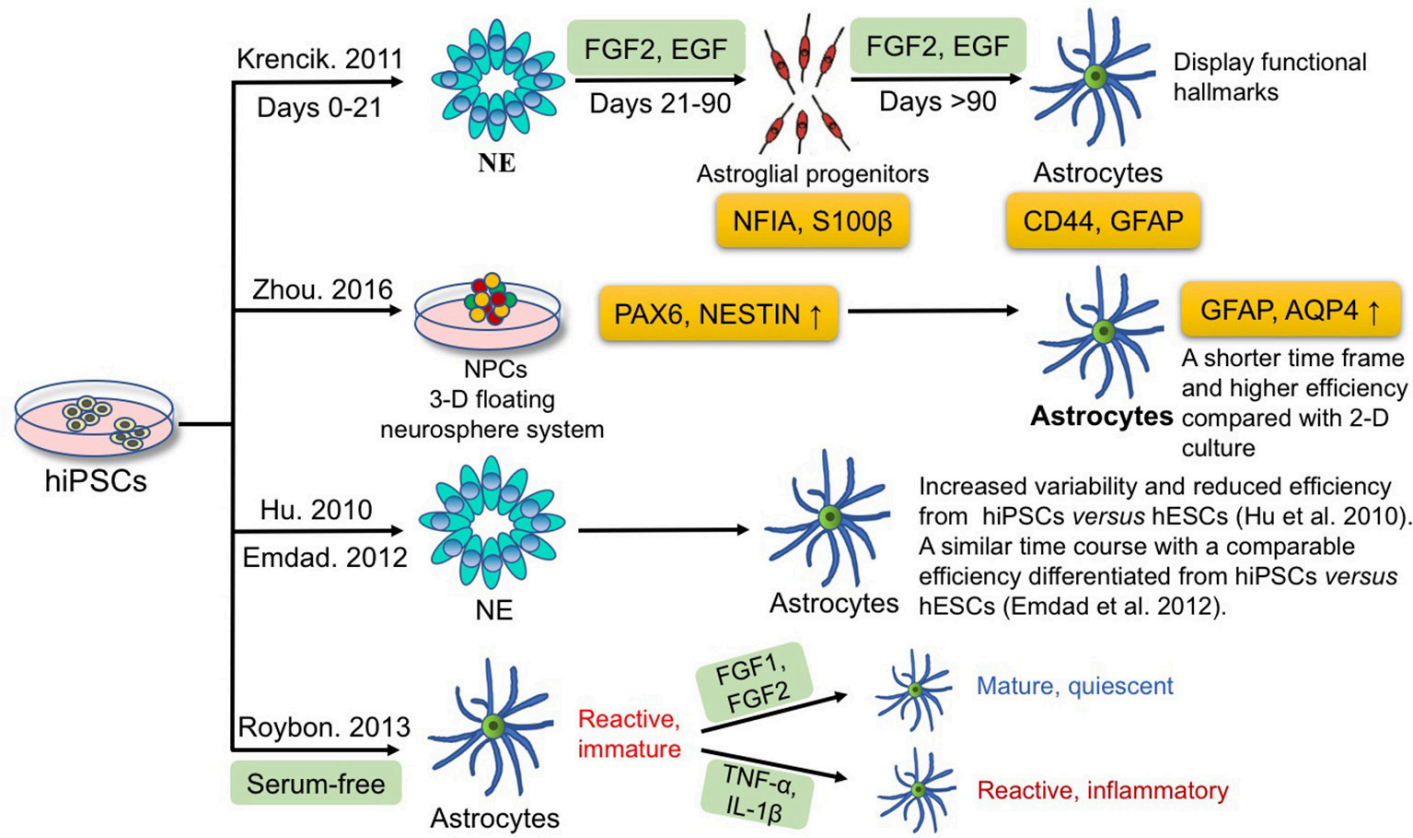
Current approaches for deriving astrocytes from hiPSCs. Krencik et al. produced astroglial progenitors and immature astrocytes differentiated from hiPSCs through a 6-month process in the presence of FGF2 and EGF. Zhou et al. employed a 3-D floating neurosphere system to increase the efficiency and to shorten the time frame of astrocytes differentiation. Hu et al. and Emdad et al. compared the time course, variability and efficiency using hiPSCs vs. hESCs for differentiation into astrocytes. Roybon et al. reported that FGF1 or FGF2 can promote maturation to a quiescence state without triggering inflammation; yet TNF-α and IL-1β have opposite effects and induce a reactive inflammatory state of astrocytes.

In the case of astrocytes, many studies have focused on the differentiation of astrocytes from hiPSCs. Figure 2 summarizes the current approaches for astrocyte differentiation from hiPSCs. A chemically defined differentiation system was first reported for efficient production of astroglial progenitors and immature astrocytes from hiPSCs in large quantities (estimated 2.8 × 10^12^ immature astrocytes) through a 6-month long term expansion process in the presence of FGF2 and epidermal growth factor (EGF) (Krencik et al., 2011). In detail, astrocyte differentiation includes three major stages; hiPSCs are first differentiated into neuroepithelial cells (days 0–21), then to astroglial progenitors with addition of EGF and FGF2 in the medium (days 21–90), and finally immature astrocytes are generated (days >90). Astroglial progenitors and immature astrocytes express NFIA-S100β at 4–8 weeks, and express more mature markers CD44-GFAP by 8–12 weeks of differentiation. Besides, these hiPSC-derived astrocytes display functional hallmarks that include possessing glutamate receptors and transporters, propagating calcium waves, promoting synaptogenesis, and taking part in the formation of the blood-brain barrier (Krencik et al., 2011). Moreover, these differentiated astrocytes are not contaminated with other cell types, such as microglia, in comparison with primary astrocyte cultures (Krencik and Zhang, 2011). In the subsequent years, the period of astrocyte differentiation from iPSCs was further shortened to 35–80 days by manually picking rosettes on the initial stage of neural precursor generation (Emdad et al., 2012; Juopperi et al., 2012; Lafaille et al., 2012; Shaltouki et al., 2013). Since iPSCs have been confirmed to be able to differentiate to astrocytes, the next question is how to optimize the differentiation process. Compared with traditional two-dimensional (2-D) adherent culture, Zhou et al. employed a 3-D floating neurosphere system to increase the yield of neural progenitors positive for PAX6 and NESTIN, compared with 2-D adherent monolayer culture, which subsequently improved the efficiency of astrocyte generation from iPSCs, as monitored by GFAP and aquaporin 4 expression (Zhou et al., 2016). On the other hand, reliable 3-D-based culture methods, have proved to be able to accelerate astrocyte differentiation, and shorten the time frame, compared with the traditional 2-D method. Furthermore, human embryonic stem cells (hESCs) have been demonstrated to differentiate to neurons and glia in a chemically defined culture (Li et al., 2005; Watanabe et al., 2007; Yang et al., 2008). Hu et al. compared the neural-differentiation capacity between hiPSCs and hESCs and found hiPSCs differentiate to neuroepithelia and functional neurons and glia over similar temporal patterns as hESCs do but with increased variability and reduced efficiency (Hu et al., 2010). The results indicate that not only techniques are needed to improve the differentiation potency of iPSCs, but better criteria are also needed to select more uniform iPSCs. Later, Emdad et al. utilized two hESC and two hiPSC lines and specified them to neuro-epithelial lineages and then to astrocytes (Emdad et al., 2012). They found that hiPSCs are differentiated to astrocytes in a similar time course and with a comparable efficiency vs. hESCs (Emdad et al., 2012).

Actually, a complex interaction between intrinsic gene expression and extrinsic environmental factors regulates neurogenesis and gliogenesis during embryonic development. It is necessary to take intrinsic and extrinsic factors into consideration, such as reprogramming transgenes in iPSCs, culture conditions, and differentiation protocols, etc. Nevertheless, most existing protocols yield only immature astrocytes and the phenotypes of astrocytes are extremely dynamic. For instance, ischemia and pro-inflammatory factors can induce reactive astrocytes, which can be further categorized into A1 (harmful) and A2 (helpful) astrocytes (Liddelow and Barres, 2017; Liddelow et al., 2017). From the application standpoint, quiescent mature astrocytes are essential for modeling normal development and functions whereas reactive astrocytes are conducive to studies involving the modeling of human pathological processes *in vitro*. Indeed, GFAP^+^ astrocytes obtained through standard protocols are of immature reactive phenotypes, marked by high GFAP and low glutamate transporter 1 (GLT1) expression compared with quiescent state which shows high GLT1 and low GFAP expression (Zamanian et al., 2012). Therefore, it is important to develop a robust method to generate mature, quiescent astrocytes, so as to facilitate a better understanding of astrocyte function in health and disease. Roybon et al. reported that FGF1 or FGF2 can promote maturation to a quiescence state without triggering inflammation; yet TNF-α and IL-1β have opposite effect and induce a reactive inflammatory state of astrocytes (Roybon et al., 2013).

### OLs

**Figure 3:**
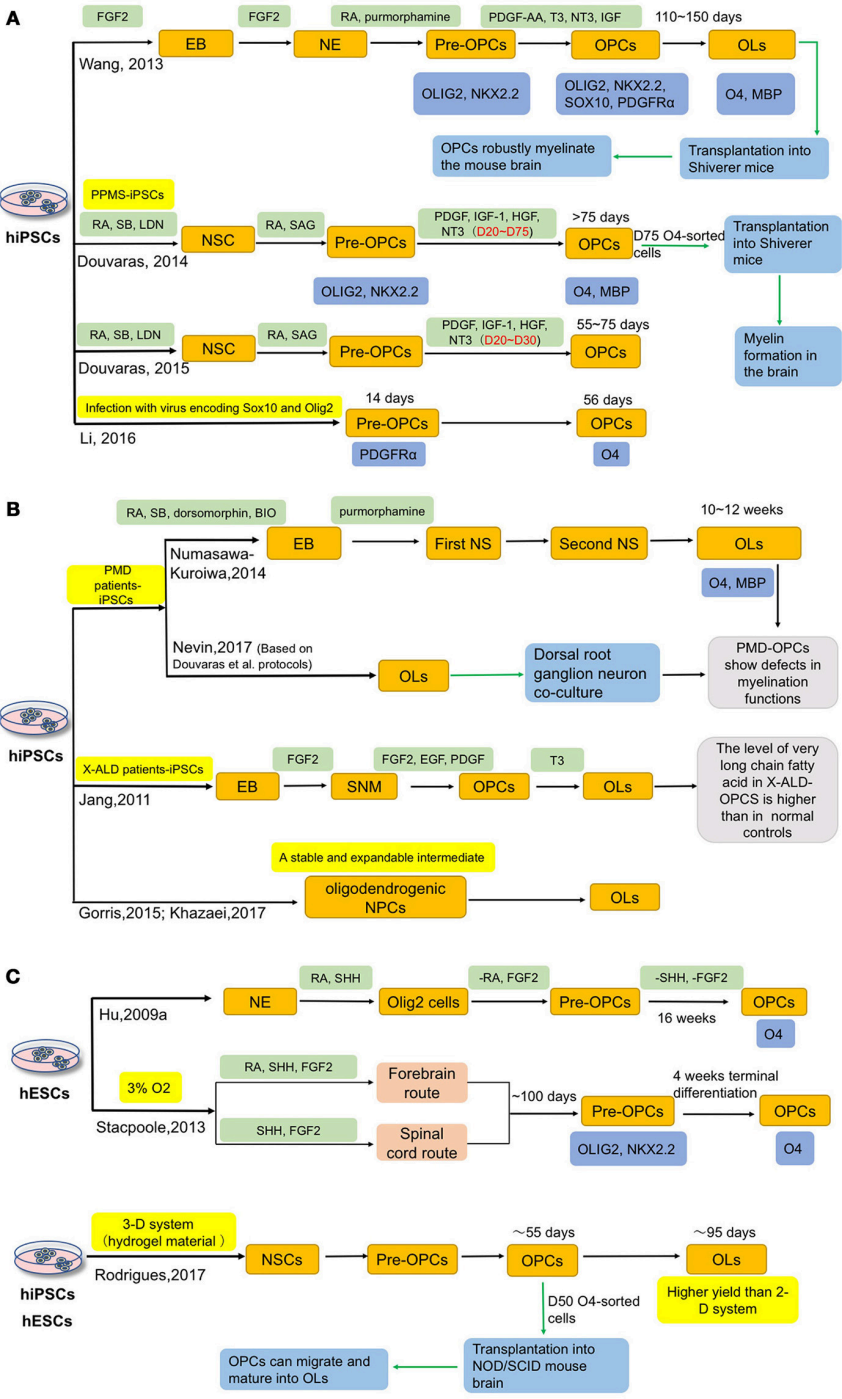
Current approaches for deriving OLs from hiPSCs. Oligodendrocytes can be induced from both hESCs and hiPSCs. **(A)** Wang, Douvaras and Li et al. induced hiPSCs, including MS patient-iPSCs, intoOPCs, respectively. Besides, they accelerated the process by adding different small molecules or infecting virus. **(B)** In order to better understand the role of OPCs in related diseases, several groups have differentiated patient-iPSCs into OPCs, such as Pelizaeus-Merzbacher disease (PMD), X-linked adrenoleukodystrophy (X-ALD). Additionally, generation of a stable and expandable intermediate, oligodendrogenic NPCs, could provide a convenience advantage for potential clinical applications. **(C)** In 2009, Hu et al. used SHH as a key factor to induce OLs from hESCs. And Stacpoole et al. shortened the differentiation period to 120 days by inducing OPCs in a low oxygen tension environment. Compared with the traditional 2-D culture system, Rodrigues et al. successfully generated hESCs and hiPSCs into OPCs in 3-D systems, which could get higher yields of OPCs and may also provide a platform to study the cell-cell interactions *in vitro*.

OLs have a powerful myelinating capacity and have become the optimal cell source for modeling and treating demyelinating diseases. Figure 3 summarizes the current approaches for differentiation of OLs from hiPSCs. Hu et al. used SHH, both a ventralizing morphogen and a mitogen (Goldman and Kuypers, 2015), as a key factor to induce OLs from hESCs (Hu B. Y. et al., 2009). After 16 weeks of differentiation, O4^+^ OLs can be obtained and reached around 40% of all cells (Hu B. Y. et al., 2009). After that, more protocols were reported (Hatch et al., 2009; Hu Z. et al., 2009; Stacpoole et al., 2013; Wang et al., 2013; Major et al., 2017).

Compared with hESCs, hiPSCs possess certain advantages and raise fewer ethical and transplantation-associated immunological problems. In 2012, Song et al. reported that skin cells-derived iPSCs from MS patient could be successfully differentiated into OLs, astrocytes and neurons (Song et al., 2012). This study provided a new approach to MS research. To assess the myelinating capacity, Wang et al. developed a 6-stage strategy by adding different small molecules at different stages and transplanted iPSCs-derived OPCs into shiverer mice (a mouse model deficient in myelination) (Wang et al., 2013). Three healthy people-derived iPSC lines were differentiated into OPCs, which took 110–150 days, with a high efficiency of around 80% (Wang et al., 2013). These OPCs can produce myelin basic protein (MBP) when co-cultured with human axons; after transplantation into newborn shiverer mice, the OPCs robustly myelinate the brain and significantly improve the survival rate of the mice (Wang et al., 2013). Importantly, there is no sign of tumor formation 9 months post-transplantation (Wang et al., 2013). Although this protocol could achieve a higher OPC production than previous methods, it is still a lengthy process. A more efficient protocol that takes less time would be favorable. To meet this need, Stacpoole et al. showed that OPCs could be obtained in a shorter period of time with a high yield by inducing them in a low oxygen tension environment (Stacpoole et al., 2013). After 120 days, 87% of total cells are Olig2^+^/NG2^+^ double positive, and a few cells express O4; and following another week, the proportion of O4^+^ cells increase to 46% (Stacpoole et al., 2013). Douvaras et al. applied dual SMAD inhibition for neural induction under an adherent condition and determined the optimal concentration of retinoic acid (RA) and successfully differentiated OLs from primary progressive multiple sclerosis patients (PPMS)-derived iPSCs (Douvaras et al., 2014). With this method, 28–80% O4^+^ OPCs can be generated within 75 days and O4-sorted cells have the capacity of myelination in shiverer mice (Douvaras et al., 2014). They suggested that PPMS-derived OPCs have equal functions to those derived from healthy subjects (Douvaras et al., 2014). In their follow-up studies, the differentiation time to obtain O4^+^ cells is further shortened from 75 to 55 days (Douvaras and Fossati, 2015). Later on, Li et al. applied a concept of “forward patterning” to accelerate the OPC differentiation from iPSCs by a forced expression of Sox10 and/or Olig2 in iPSCs. In this way, they can obtain PDGFRα+ OPCs in 14 days and O4+ OPCs in 56 days (Li et al., 2016). Additionally, to accelerate the differentiation to OPCs and OL2, addition of small molecules, such as clemastine and benzatropine (Mei et al., 2014), may be considered.

In addition to MS, Pelizaeus-Merzbacher disease (PMD) and X-linked adrenoleukodystrophy (X-ALD) are two diseases in which OLs are closely involved. Several groups have independently generated OLs from PMD and X-ALD patients-specific iPSCs (Jang et al., 2011; Numasawa-Kuroiwa et al., 2014; Nevin et al., 2017). Numasawa-Kuroiwa et al. achieved OPC differentiation by first inducing iPSCs into embryoid bodies (EBs) and then neurospheres using small molecules that inhibit dual SMAD and GSK3 pathways. After 10–12 weeks of differentiation, MBP^+^ mature OLs appear (Numasawa-Kuroiwa et al., 2014). Later on, Nevin et al. differentiated OPCs from 12 PMD iPSC lines and 7 control iPSC lines, using the protocol developed by Douvaras et al. (Douvaras et al., 2014; Douvaras and Fossati, 2015; Nevin et al., 2017). PMD patients-specific iPSCs-derived OPCs exhibit defects in process extension and branching *in vitro* and *in vivo* (Numasawa-Kuroiwa et al., 2014; Nevin et al., 2017). Jang et al. induced X-ALD patients-specific iPSCs into spherical neural masses (SNMs), then differentiated SNMs to OPCs by treatment with several small molecules (Jang et al., 2011). This is the first report of X-ALD iPSCs-derived OPCs, and the authors found that the level of very long chain fatty acid in X-ALD OPCs is significantly higher than in normal controls (Jang et al., 2011).

In addition to these studies, other methods have also been reported. Gorris et al. differentiated iPSCs to an intermediate neural progenitor cells (NPCs) state that are predisposed to oligodendrocyte fate specification (oligodendrogenic NPCs). The oligodendrogenic NPCs can be stocked and frozen for future use—a convenience advantage for potential clinical application (Gorris et al., 2015; Khazaei et al., 2017).

A 2-D cell culture system was used in the above studies, which shows limitations in mimicking the cell-cell interactions in human brain. To address this issue, researchers have developed 3-D culture systems for OPC differentiation (Egawa et al., 2017; Rodrigues et al., 2017). Egawa et al. developed a 3-D system using gels composed of a mixture of collagen and hyaluronan, in which rat primary OPCs can proliferate and differentiate into mature OLs (Egawa et al., 2017). In the study of Rodrigues et al., OPCs are generated in a 3-D system based on a hydrogel material. Three hESC lines and one iPSC line are induced to OPCs by using the dual-SMAD inhibitors (SB431542 and LDN193189), SAG, and RA; O4^+^ cells appear on day 55, and then the MBP^+^ OLs are produced on day 95 with a higher yield than with a 2-D culture system. To evaluate the functions of OPCs, the authors injected the 50-day-old cells into NOD/SCID (non-obese diabetic-severe combined immunodeficient) mouse brains and found these cells can proliferate and migrate *in vivo* after 6 months. Moreover, the OPCs generated in the 3-D culture also have spiking behaviors (Rodrigues et al., 2017).

The current differentiation methods all give rise to a heterogeneous population consisting of not only OLs, but also other cell types, such as astrocytes and neurons. O4-sorted OPCs have to be engrafted immediately, or cultured together with other cells for expansion or for functional analysis; sorted OPCs cannot survive passaging as a pure population. Future studies are required to determine a culture condition in which sorted OPCs can be further expanded.

### Microglia

**Figure 4:**
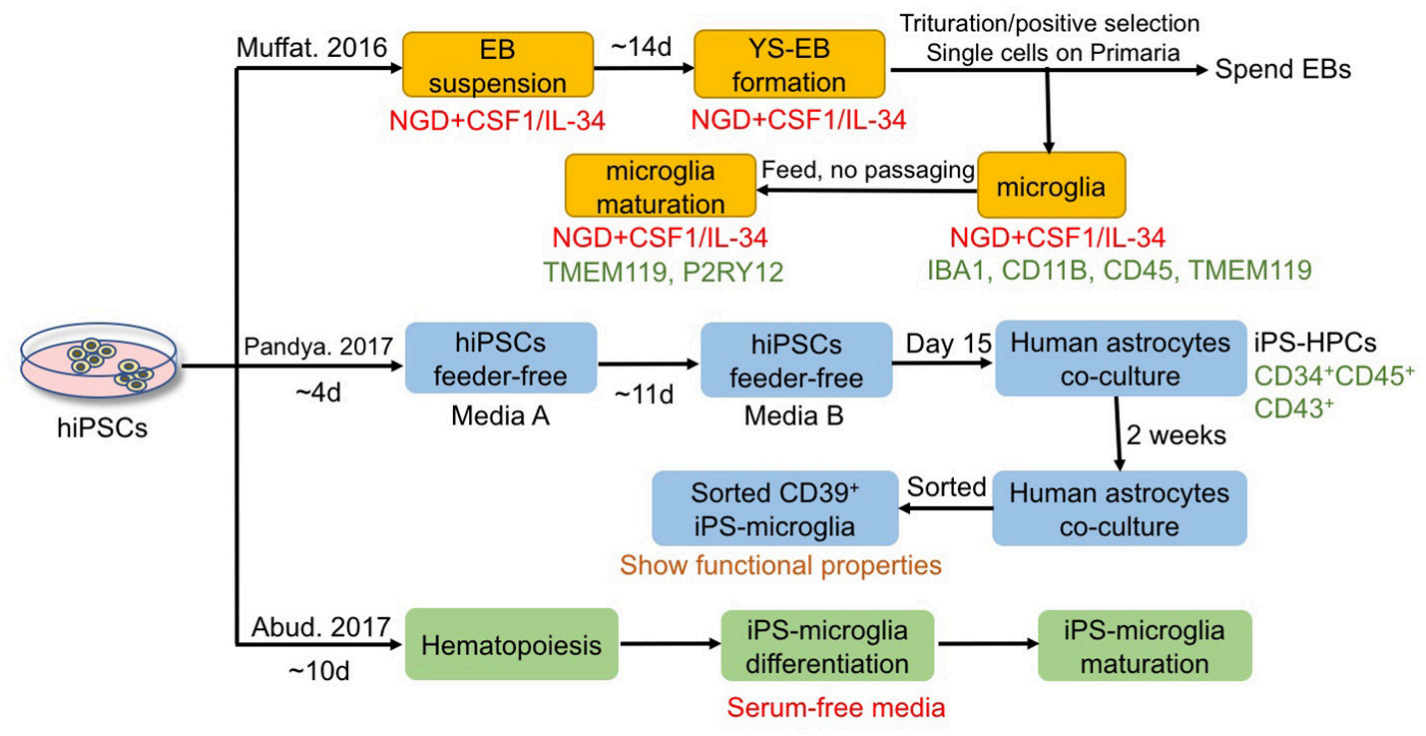
Current approaches for deriving microglia from hiPSCs. Muffat et al. demonstrated that hiPSCs can be differentiated to microglia with neuroglial differentiation (NGD) media in the presence of CSF1 and IL-34, and the obtained cells possess the functions of phagocytes positive for microglial markers such as TMEM119 and P2RY12. Pandya et al. reported another method that applies co-culture with astrocytes for differentiation of hiPSCs to microglia-like cells via a hematopoietic progenitor-like intermediate stage. And Abud et al. established a fully defined and serum-free method to derive microglia-like cells from hiPSCs with robust scalability and high purity, via differentiating first toward hematopoiesis stem cells and then to microglia.

Microglia have aroused more attention in recent years as significant modulators of neurogenesis, development, function, and pathogenesis of the CNS. However, similar challenges exist in microglial research, which include limited accessibility of human neural tissues, phenotype of microglia being unpredictably changed by current isolation methods, difficulties in culturing microglia *in vitro*, and the fact that murine glial cells cannot simulate all the features of primary human glial cells. One solution to overcome these hurdles is to derive microglia from hiPSCs using a chemically defined method. Although many studies have focused on the differentiation of hiPSCs to astrocytes and OLs, only very limited studies have reported successful generation of microglia from hiPSCs. Figure 4 summarizes the current approaches for microglia differentiation from hiPSCs. One of the reasons lies in the fact that microglia are derived from yolk sac myeloid precursors, which cannot be produced from neural progenitors as astrocytes and OLs can be. Nevertheless, Muffat et al. first established a protocol in which neuroglial differentiation (NGD) medium with addition of colony-stimulating factor 1 (CSF1) and IL-34 was used for generating hiPSC-derived microglia-like cells, which resemble primary fetal and human adult microglia (Muffat et al., 2016). In detail, colonies were triturated mildly to form a suspension of EBs and transferred to NGD medium (10 ng/ml CSF1 and 10 ng/ml IL-34) (Muffat et al., 2016). One of the two main types of EBs formed presented with large, expanding cystic bodies, and were called yolk sac EBs (YS-EBs). Then YS-EBs were triturated every 5 days to shear off loose cells, and EBs that were attached on Primaria plates were selected (Muffat et al., 2016). Muffat et al. also demonstrated that hiPSC-derived microglia are capable of migration and proliferation, possessing the functions of phagocytes positive for microglial markers such as IBA1, CD11B, CD45, TMEM119, and P2RY12 (Muffat et al., 2016). Besides, 20 different ES and iPSC lines have been differentiated to microglia successfully to ensure the reproducibility of the protocol, allowing for robust and efficient generation of microglia from iPSCs of different genetic backgrounds (Muffat et al., 2016). Later, Pandya et al. reported another method for differentiation of hiPSC into microglia-like cells via a hematopoietic progenitor-like intermediate stage, instead of through neural intermediate stage used for astrocyte and OPC differentiation, which is more consistent with the embryonic development *in vivo* (Pandya et al., 2017). In the protocol of Pandya et al., hiPSCs were cultured feerder-free in media A (STEMdiff APEL medium with hVEGF, hBMP4, hSCF and hActivin A) for the first 4 days (Pandya et al., 2017). Then on days 4, 7, 10, media A was changed to media B (STEMdiff APEL medium with hSCF, hFlt3L, hIL-3, hIL-6, hG-CSF, and hBMP4) (Pandya et al., 2017). On day 15, cells were harvested and plated onto human astrocytes for 1–2 weeks for differentiation into microglia (Pandya et al., 2017). The microglia-like cells show functional properties, including producing reactive oxygen species, phagocytosing cellular debris, secreting pro-inflammatory factors (Pandya et al., 2017). Microglia derived from murine iPSCs are able to increase the survival of mice with intracranial malignant gliomas (Pandya et al., 2017). In this study, the murine microglia were obtained through a similar protocol as used to generate hiPSC-derived microglia-like cells (Pandya et al., 2017). Significantly, in the published protocol, Pandya et al. utilized astrocytes differentiated from the same starting hiPSCs as feeder cells to facilitate generation of human microglia; avoiding the use of murine cells and the subsequent introduction of xenogeneic cells/proteins are advantageous for possible future clinical applications using hiPSC-derived microglia (Pandya et al., 2017). Furthermore, the Pandya method shortens the differentiation period from 8 to 4 weeks by using a co-culture system with hiPSC-astrocytes; the culture medium is also improved to a factor-defined one, instead of using a serum-based medium (Pandya et al., 2017). More recently, a highly efficient, fully defined and serum-free method was described for differentiating microglia-like cells from hiPSCs with robust scalability and high purity, via differentiating toward hematopoiesis stem cells and then to microglia (Abud et al., 2017). Whole-transcriptome analysis and functional assessment revealed that microglia-like cells derived from hiPSCs are similar to those derived from human fetal and adult tissues (Abud et al., 2017). In summary, these fully defined protocols can produce a large number of microglia-like cells, which offers a renewable source of cells to investigate the roles of microglia in normal CNS development, functions, and homeostasis, as well as the roles in various neurological diseases. However, there still exists a pressing need to further promote the yield, purity and scalability of microglia derived from hiPSCs.

Based on the critical roles of glial cells in CNS physiology, it is not surprising that glial cell dysfunction has been involved in many neurological diseases. In the next part, we will discuss recent advancements on the non-neuronal cell-autonomous effects of glia in some representative neurological diseases (summarized in Table 2), with an eye toward using iPSC-derived glial cells to create once inaccessible cell types, providing new tools to model complex neurological diseases, and developing new targets for autologous cell transplantation.

**Table 2:** Potential application of hiPSC-derived glial cells in neurodegenerative diseases and cell replacement therapy.

**Diseases involving glia**	**Cell types**	**Representative roles in disease pathology**	**Potential applications**	**Challenges**	**References**
Amyotrophic lateral sclerosis (ALS)	Astrocytes	Astrocytes from ALS patients are toxic to non-ALS derived motor neurons; selective knockdown of mSOD1 in astrocytes results in a significant rescue of astrocyte-derived toxicity; SOD1^G93A^ astrocytes can cause motor neuron pathology and death, respiratory and forelimb motor dysfunction.	mSOD1 in astrocytes can be recognized as a potential therapeutic target for ALS; transplantation of hiPSC-derived glia-rich progenitors, which then give rise to astrocytes, into the cervical or lumbar spinal cord of ALS patients improves the motor function.	Lacking standard protocols for differentiation of hiPSCs to glial precursors; allowing the long-term survival; reducing the immune responses; mitigating the risk of tumor formation as well as establishing appropriate circuitry with host CNS. Several factors may account for the cell transplantation effects for ALS, including patient selection (disease stages), injection sites (cervical or lumbar or both), and modification of cellular grafts (glial-restricted precursors with/without GLT1 overexpression).	Haidet-Phillips et al., 2011 Papadeas et al., 2011 Kondo et al., 2014 Lepore et al., 2008 Philips and Robberecht, 2011 Zhu et al., 2002 Boillée et al., 2006 Henkel et al., 2009 Tang and Le, 2016
	Microglia	Microglia are activated where motor neuron loss takes place, contributing to the death of motor neurons; Minocycline treatment delays ALS progression through inhibiting microglial activation; selective removal of mSOD1 from microglia slows down disease progression; mSOD1 may induce M2-microglia to transform into M1-microglia as disease progresses.	Antibiotics may serve as a potential therapy for ALS; mSOD1 in microglia can be recognized as a potential therapeutic target for ALS; Manipulating the balance between M1 and M2 microglial phenotypes during specific stage(s) in ALS may provide clinical therapeutic benefits.		
Spinal cord injury (SCI)	Astrocytes	Transplantation of astrocytes from murine glial-restricted progenitors significantly improves behavioral recovery and induces robust axonal growth after acute injury of rat spinal cord; transplantation of GLT-overexpressing hiPSCs-astrocytes into rodent animals reduces lesion size and promotes preservation of respiratory functions.	Strategies of transplanting astrocytes from hiPSCs-derived glial-restricted progenitors provide a potential for promoting regenerative growth of both motor and sensory axons after SCI.	Many key factors should be taken into consideration that include SCI injury models (acute, sub-acute and chronic), source of transplanted cells, methodologies of assessing the efficacy of transplanted cells and the timeframe of observation after transplantation.	Davies et al., 2006 Li et al., 2015 Nutt et al., 2013 Hayashi et al., 2011
	Oligodendrocytes (OLs)	Severe loss of OLs leads to demyelination, and subsequently activation of OPC proliferation, which would last for about 1 week. However, ER stress and negative regulators (BMP-4, semaphorin 3 etc.) at the injury sites inhibit the rescue effects of OPCs.	Screening drugs that are neuroprotective and/or able to promote maturation and remyelination of OPCs in the lesioned cord; testing the optimal cell dosage and time points after SCI; combining cellular grafts with drug treatment for SCI, such as clemastine and benzaltolide etc.	Optimal implantation time window; accurate animal models; limitation of transplanting a single type of cells; survival, maturation, and remyelination of donor OPCs in host cord.	Zai and Wrathall, 2005; Lytle et al., 2009; Xiao et al., 2010; Jang et al., 2011; Mei et al., 2014; Imai et al., 2018
Alzheimer's disease (AD)	Microglia	Microglia recruitment serves as part of the attempt of the CNS to clear Aβ deposition, which plays a neuroprotective role at the early stage of AD; but as disease progresses, microglia become dysfunctional and pro-inflammatory cytokines released by microglia downregulate genes involved in Aβ clearance pathways, therefore contributing to Aβ accumulation and neurodegeneration at the late stage of AD.	Inflammatory processes, including activated microglia accumulation, have been demonstrated to contribute to neuronal damage in AD, suggesting anti-inflammatory strategies may offer some neuroprotection.	A previous clinical trial reported negative results from prednisone treatment in AD patients. But the anti-inflammatory strategy for AD treatment should not be nullified. Additional endpoints, more anti-inflammatory drugs of various doses, and other inflammation biomarkers should be tested in future trials.	El Khoury et al., 2007 Hickman et al., 2008 Liu et al., 2010 Tang and Le, 2016 Koch and Szecsey, 2000
Multiple Sclerosis (MS)	OLs	OPCs could restore part of the myelin damage during the remission phase; but in the progressive phase, endogenous OPCs fail to rescue the demyelinated axons, which ultimately leads to neurological function deficits.	Transplanted hiPSC-OPCs into EAE models at different phases of disease to evaluate the differences in therapeutic effect; combining cell transplantation with drug treatment for treatment of MS, such as clemastine, benzaltolide and/or rHIgM22 etc.	Survival, maturation and remyelination of donor OPCs in host cord; insufficiency of animal models to reflect human diseases.	Bjartmar and Trapp, 2001 Pirko et al., 2004 Kuhlmann et al., 2008 Tallantyre et al., 2010 Boyd et al., 2013 Mei et al., 2014 Thiruvalluvan et al., 2016

## Potential application of hiPSC-derived glial cells in neurological diseases and cell replacement therapy

### Amyotrophic lateral sclerosis

ALS is an adult-onset disorder that is characterized by rapid selective upper or lower motor neuron degeneration, resulting in hyperreflexia, spasticity (upper motor neurons), muscle weakness, and paralysis (lower motor neurons) and ultimately fatal respiratory failure (Rowland and Shneider, 2001; Kiernan et al., 2011). About 5–10% of classical ALS cases are familial, and the remaining sporadic (Brown, 1997). With regard to the mechanism of ALS, mutations in the Cu/Zn superoxide dismutase 1 (SOD1) gene have been identified as the main cause of ~20% familial ALS (Brown, 1997; Bruijn et al., 2004). In spite of much effort that has been made in the past two decades, there is still no effective therapy to slow down the disease progression. Besides, modeling ALS with transgenic rodent animals is costly and time-consuming since ALS is a slowly developing neurodegenerative disorder, requiring months of modeling using transgenic rodent animals. Therefore, utilizing hiPSC-based *in vitro* models of ALS has attracted interest in the research community. Some studies have emphasized the role of neuroglia in the pathogenesis of ALS. Astrocytes derived from both sporadic ALS patients and familial ALS patients have been demonstrated to be toxic to non-ALS derived motor neurons, suggesting that sporadic ALS and familial ALS may share a similar mechanism through astrocytes-mediated cellular toxicity (Haidet-Phillips et al., 2011). And knockdown of mutant SOD1 in astrocytes results in a significant rescue of astrocyte-derived toxicity toward motor neurons, indicating that mutant SOD1 in astrocytes can be recognized as a potential therapeutic target for ALS (Haidet-Phillips et al., 2011). Furthermore, Bi et al. has demonstrated that lipocalin as a potent mediator of astrocytic toxicity is secreted by reactive astrocytes and is highly selectively toxic to neurons, especially to the unhealthy neurons which express ALS related genes, such as mutant TAR DNA-binding protein 43 and RNA-binding protein fused in sarcoma (Bi et al., 2013). To explore whether astrocyte-specific influences on motor neurons in ALS can translate *in vivo*, Papadeas et al. transplanted glial-restricted precursors harboring the human G93A mutation (SOD1^G93A^) into the spinal cord of wild type rats, and the donor cells differentiated into astrocytes in rats eventually (Papadeas et al., 2011). The results showed that SOD1^G93A^ astrocytes can cause motor neuron pathology and death, respiratory and forelimb motor dysfunction, as well as a reduced GLT1 transporter expression in rats (Papadeas et al., 2011). Moreover, targeted enrichment of normal astrocytes into mutant SOD1 rats to facilitate restoration of motor neuron function has been investigated by Kondo et al. (Kondo et al., 2014). Transplantation of hiPSC-derived glia-rich progenitors, which then gave rise to astrocytes, into the lumbar spinal cord of ALS mice improved the motor function and prolonged the lifespan of mice via activation of AKT signals (Kondo et al., 2014), exemplifying the rationale of cell therapy for ALS using hiPSC-derived glial cells. Consistent findings were also observed when glial-restricted precursors were transplanted into the cervical spinal cord of 90-day old SOD1^G93A^ rats (Lepore et al., 2008). Glial-restricted precursors survived, attenuated motor neuron death, and extended the lifespan of animals (Lepore et al., 2008). These data support that astrocyte dysfunction may serve as a propagator of motor neuron degeneration in ALS, and astrocytes may play critical roles in SOD1 mutation-mediated ALS, although it remains unknown whether astrocyte dysfunction is a driver or consequence of ALS. Based on the evidence that astrocytes-derived toxicity plays an active role in motor neuron death in ALS, astrocytes may represent a potential therapeutic target in ALS; replacement of dysfunctional astrocytes or enrichment of normal astrocytes with functioning astrocytes, may serve as one potential approach in ALS therapy. With respect to clinical trials, an open label phase I/IIa study (NCT03482050), aimed to evaluate the safety, tolerability and therapeutic effects of astrocyte transplant derived from hESCs to treat ALS patients, is recruiting participants at present. In this trial, astrocytes derived from hESCs will be administered through intrathecal (spinal) injection with an escalating low, medium, and high dose or two consecutive administrations of medium dose with an interval. The study will test the hypothesis that transplantation of astrocytes may compensate for the patients' own dysfunctional astrocytes via restoring the normal physiological functions that include, for example, reducing oxidative stress and other toxic compounds as well as regulating extracellular neurotransmitter levels. This study will be the first to apply stem cells-derived astrocyte graft into patients with ALS, accelerating the translation of stem cell biology to clinical applications to treat neurological diseases. Besides, another clinical trial (NCT02943850), aimed to examine the safety of transplanting GDNF-producing NPCs into the spinal cord of ALS patients, is recruiting participants now. This trial is a single-center, Phase 1/2a study using two escalating doses of human NPCs expressing GDNF, which will be injected unilaterally into the lumbar region of ALS patients with moderate leg involvement. This is the first trial to use genetically modified progenitors to treat ALS.

Microglia have also been identified as a critical player in ALS progression. Microglia are activated in areas where motor neuron loss takes place, contributing to the death of motor neurons (Philips and Robberecht, 2011). Nuclear factor-kappa B (NF-κB) pathway, a mediator of inflammation, was suggested as a possible mechanism of microglia-mediated motor neuron death (Frakes et al., 2014). Minocycline, a second-generation tetracycline, is capable of delaying ALS progression and extending the life span of ALS mice through inhibiting microglial activation, suggesting that certain antibiotics may serve as a potential drug for ALS via inhibiting microglial activation (Yrjänheikki et al., 1999; Zhu et al., 2002). Microglia isolated from mutant SOD1^G93A^ transgenic mice and activated by LPS treatment are more neurotoxic to motor neurons compared with wild-type microglia (Xiao et al., 2007). Furthermore, Boillée et al. found that removal of mutant SOD1 gene from microglia had little effect on the early disease onset whereas slowed down disease progression during the late disease phase, indicating that mSOD1 in microglia can be recognized as a potential therapeutic target for ALS (Boillée et al., 2006). Interestingly, M1 and M2 microglial phenotypes may switch during ALS disease development. Intracellular and extracellular misfolded mutant SOD1 may play critical roles in regulating the transformation from anti-inflammatory M2 phenotype to pro-inflammatory M1 phenotype as disease progresses, through promoting the generation and release of reactive oxygen species and exaggerating pro-inflammatory signaling in microglia (Henkel et al., 2009; Tang and Le, 2016). Collectively, while the M2-like microglia appear to be protective in the first response, the following sustained neuro-inflammatory cytokine cascade and neuronal stress exerted by mutant SOD1 induce microglia to transform into M1-like phenotype. From a therapeutic perspective, manipulating the balance between M1 and M2 microglial phenotypes during specific stage(s) in neurodegenerative diseases may provide clinical therapeutic benefits. In conclusion, the cellular pathogenesis of ALS is not limited to motor neuron abnormalities but extend to glial cells, such as astrocytes and microglia. These studies suggest that transplantation of hiPSCs-derived glial precursor cells, which are promising candidates for cell replacement therapy due to their efficient glial restricted differentiation, low risk of tumorigenesis, and good survival capacity, may improve the microenvironment around motor neurons in ALS. However, there is still a long way to go before we can have a complete picture of how microglia and astrocytes contribute to ALS, and how we can efficiently employ hiPSCs-derived microglia and astrocytes for treatment of ALS as a cell-based therapy. Challenges include: a lack of standard protocols for differentiation of hiPSCs to glial precursors; immune responses caused by allogeneic glial grafts; how to eliminate the risk of tumor formation; and how to establish appropriate (and avoid inappropriate) circuitry with host cells. Furthermore, several factors may account for the cell transplantation efficacy for ALS, including selection of patients (genetic background affects efficacy?), time window of intervention (early or late stage?), injection sites (cervical or lumbar or both?), etc.

### Spinal cord injury

SCI is a devastating condition in which injury to spinal cord leads to extensive neural damage and severe motor or sensory functional deficits (Holmes, 2017). A large body of literature has focused on hiPSC-based transplantation strategies for promoting regenerative growth of both motor and sensory axons after SCI (Nori et al., 2011; Fujimoto et al., 2012). Transplantation of astrocytes from murine glial-restricted progenitors significantly improves behavioral recovery and induces robust axonal growth after acute injury of rat spinal cord (Davies et al., 2006). A cascade of downstream mechanisms termed “secondary injury” follows the initial injury-mediated axotomy of passing axons and cell death in SCI, including glutamate excitotoxicity caused by dysfunction of extracellular glutamate homeostasis (Park et al., 2004). Therefore, Li et al. engineered hiPSC-derived astrocytes with lentiviral vectors encoding GLT1 and transplanted these cells into rodent animals (Li et al., 2015). GLT1-overexpressing hiPSCs-astrocytes reduce lesion size and promote preservation of diaphragmatic respiratory functions of SCI model animals compared with unmodified hiPSCs-astrocytes and human fibroblast control group (Li et al., 2015). Nevertheless, Nutt et al. examined the potential of caudalized hiPSC-derived NPCs to repair early chronically (4 weeks post-injury) injured spinal cord (Nutt et al., 2013), and found that caudalized hiPSC-derived NPCs develop into neurons and glia which survive for up to 2 months but without significant restoration of forelimb functions (Nutt et al., 2013). Moreover, Hayashi et al. utilized mouse iPSCs to differentiate to astrocytes and transplanted then into rats 3 or 7 days after spinal injury (Hayashi et al., 2011). The results revealed that the animal groups injected with iPSC-derived astrocytes do not show remarkable improvement and even display greater sensitivity to mechanical stimulus than control group treated with Dulbecco's Modified Eagle Medium (Hayashi et al., 2011). These findings suggest that many key factors should be taken into consideration that include SCI injury models (acute, sub-acute, and chronic), source of transplanted cells, methodologies of assessing the efficacy of transplanted cells and the timeframe of observation after transplantation. For example, it is possible that recovery may take longer time in the chronic injury model than in the acute or sub-acute phase, and it is also reasonable that endogenous repair mechanisms have less influence in the chronic injury setting than in the acute or sub-acute model.

SCI could also cause severe loss of OLs, leading to demyelination. As a result, OPCs would be activated and respond rapidly to SCI (Crawford et al., 2014). The rate of OPC proliferation remarkably increases by day 1 after SCI and last for about 1 week, then decrease gradually (Zai and Wrathall, 2005; Lytle et al., 2009). However, the hostile microenvironment of the injury sites plays a detrimental role to their rescue effects, for instance, the existence of negative regulators that include chondroitin sulfate proteoglycans, semaphorin 3, leucine-rich repeat and Ig domain-containing 1, BMP-4 etc. (Fawcett and Asher, 1999; Ji et al., 2006; Xiao et al., 2010), and the formation of glial scars and cavity caused by astrogliosis (Jang et al., 2011). In the meantime, endoplasmic reticulum (ER) stress could also lead to apoptosis of OPCs (Imai et al., 2018). To date, therapeutic strategies to treat SCI have shown limited efficacy in clinics, and even spinal decompression can be only applied to select cases (Furlan et al., 2016; Goldman, 2018). Due to the lack of effective therapies for spinal cord injuries, transplantation of hiPSC-derived OPCs becomes a potential treatment. In 2015, All et al. transplanted hiPSC-derived OPCs into a moderate contusive SCI model in rats 24 h after injury (All et al., 2015). In this study, all rats were treated with cyclosporine A daily 3 days before cell transplantation until the end of the experiment (All et al., 2015). After 2 months, compared with the control group, the OPCs-treated group revealed the survival and differentiation of hiPSC-derived OPCs, less cavitation, more remyelination of axons, and improvement of motor behaviors (All et al., 2015). Importantly, no tumor formation was detected (All et al., 2015). This study suggests that transplantation of cells 24 h after SCI can reduce astrogliosis and inflammation (All et al., 2015). A different time point of transplantation was tested by Kawabata et al. In this study, the authors injected hiPSCs-OPCs enriched neural stem/progenitor cells (hiPSCs-OPC-enriched NS/PCs) into the lesion epicenter of T10 level in NOD-SCID mice on the 9th day after injury (Kawabata et al., 2016). Twelve weeks later, the grafted cells migrated out of the lesion site and differentiated into mature OLs, astrocytes and neurons, and these cells contributed to remyelination, functional recovery, and synapse formation between host neurons and grafted cells (Kawabata et al., 2016). In their previous studies, hiPSCs-neural stem/progenitor cells were transplanted but with an inefficient generation of OLs *in vivo*; accordingly, there was no significant improvement of motor functions after transplantation (Nori et al., 2011, 2015). To evaluate the effects of iPSCs-based therapy in immunocompetent animals, Pomeshchik et al. used tacrolimus-immunosuppressed C57BL/6J mice to test the effects of hiPSC-derived NPCs in SCI (Pomeshchik et al., 2015). However, the results showed that the transplanted cells did not lead to functional recovery (Pomeshchik et al., 2015).

In light of the limitations of rodent animals in modeling human diseases, large animal models are needed for safety and efficacy examinations prior to any possible clinical trials in human. Kobayashi et al. conducted the transplantation experiments in a non-human primate SCI model (Kobayashi et al., 2012). Twelve weeks after engraftment of hiPSCs-neural stem/progenitor cells into the fifth cervical level (C5) of spinal cord, the grafted cells show positive effects in both tissue histology and functional recovery (Kobayashi et al., 2012). In another recent study, Tuszynski and colleagues grafted human spinal cord–derived NPCs into nine adult male rhesus monkeys, which had received right-side hemisection lesions at C7 segment (Rosenzweig et al., 2018). Five successfully grafted subjects showed that human NPCs survived in the lesion sites for at least 9 months without development of teratoma or tumor; OLs and mature astrocytes were detected 5 months post-grafting, and large numbers of axons developed from grafts into distant areas of host spinal cord. Meanwhile, improvements of forelimb functions were detected in the primate models (Rosenzweig et al., 2018). Besides, the authors identified critical steps to achieve successful grafting into large lesion cavities, which included draining the CSF at the lesion site and applying fibrin–thrombin in the grafting mixture (Rosenzweig et al., 2018).

Caution needs to be taken when interpreting the results. In most of the xeno-transplantation studies using hiPSC derivatives or human fetal tissues/cells as donor cells, immunosuppressant drugs were administered. The observed positive effects, such as reduced inflammation, inhibited scar tissue formation, decreased gliogenesis, undetectable tumor formation, etc. may also result from effects of immunosuppression *per se*. Clinical trials are needed to address these issues. Since 2015, a phase I/II clinical trial has been initiated, which was aimed to evaluate the safety of dose escalation of AST-OPC1 in SCI patients (NCT02302157). AST-OPC1, an early-stage OPC population differentiated from hESCs, were used as donor cells. The safety and efficacy of AST-OPC1 had been tested in rodent SCI models, and the biological activities, biodistribution, toxicology, and tumorigenicity of the grafts had been assessed (Priest et al., 2015).

Transplantation of OPCs for SCI treatment has been contemplated since long ago; however, many challenges still exist in the field. For example, how to increase the survival of grafts; how to promote maturation and myelination of donor OPCs in host cord; what is the optimal implantation time window after SCI, etc. To enhance the efficacy of OPC transplantation, Imai et al. demonstrated that treatment with amiloride promotes OPC survival and remyelination after SCI in rats (Imai et al., 2018). Eight FDA-approved antimuscarinic compounds such as clemastine and benzaltolide could also enhance OL differentiation and remyelination (Mei et al., 2014). Based on their findings, a combinatory approach applying small molecules together with cellular grafts may be a promising strategy, which warrants further effort. In addition, SCI is often associated with large cavity in the lesioned areas; implanting OPC cellular graft alone may not be sufficient to re-bridge the sectioned connection. Adding synapse-forming neuronal precursors as a relay, with myelin-forming OPCs, may possibly achieve a better therapeutic result.

### Alzheimer's disease

As the most common cause of dementia, AD is a neurodegenerative disorder characterized by increasing memory loss, personality changes and finally cognitive impairment (Bateman et al., 2012). The accumulation of amyloid β (Aβ), and tau hyperphosphorylation, two pathological hallmarks of AD, lead to synaptic and neuronal loss as well as neuroinflammation (Bateman et al., 2012; Jack et al., 2013). Aβ aggregates can attract and induce activation of primary microglia. As the resident immune cells of the CNS, microglia have been demonstrated to be able to phagocytose amyloid fibrils; and microglia dysfunction could accelerate Aβ aggregates, leading to neurodegeneration and progression of AD (El Khoury et al., 2007; Johansson et al., 2015). Emerging evidence shows that microglia play a protective role in AD progression via clearing Aβ deposition. However, the roles of microglia in AD may be a double-edged sword, which should be taken into account when contemplating microglial or anti-inflammatory therapy for AD (Hickman et al., 2008; Tang and Le, 2016). On the one hand, microglia recruitment serves as part of the attempt of the CNS to clear Aβ deposition, which plays a neuroprotective role at the early stage of AD (El Khoury et al., 2007; Hickman et al., 2008; Liu et al., 2010). On the other hand, as disease progresses, microglia become dysfunctional and pro-inflammatory cytokines released by microglia downregulate genes involved in Aβ clearance pathways, including Aβ-binding receptors and Aβ-degrading enzymes, therefore contributing to Aβ accumulation and neurodegeneration at the late stage of AD (El Khoury et al., 2007; Hickman et al., 2008). Abud et al. transplanted hiPSC-derived microglia-like cells (day 38 of differentiation) into the cortex of mice, and found that these microglia-like cells survived, displayed highly branched morphology and expressed markers such as IBA1, TMEM119, and P2RY12 (Abud et al., 2017). Moreover, microglia-like cells derived from hiPSCs were transplanted into the hippocampi of AD mice to examine their roles in AD pathology (Abud et al., 2017). hiPSC-derived microglia-like cells migrated toward Aβ deposition and phagocytosed fibrillar Aβ as well as tau protein (Abud et al., 2017), providing a platform where hiPSC-derived microglia-like cells can be used to investigate microglial functions in AD pathology and therapy. Furthermore, genome-wide association studies have revealed that several genes expressed by microglia are involved in the regulation of neuro-inflammation in AD progression, such as *CD33* and *TREM2* (Villegas-Llerena et al., 2016). And inflammatory processes, including accumulation of activated microglia, have been demonstrated to contribute to neuronal damage in AD, suggesting that anti-inflammatory intervention may offer some neuroprotection (Tang and Le, 2016). A great number of laboratory and epidemiologic studies support that anti-inflammatory drugs decrease the incidence of AD to some extent (McGeer et al., 1996). However, a randomized, placebo-controlled multicenter trial indicates that low dose prednisone used in the study was ineffective in slowing the rate of cognitive decline in AD patients (Koch and Szecsey, 2000). There was no significant difference in cognitive decline between the prednisone and placebo treatment groups, as measured by the 1-year change in AD Assessment Scale (Koch and Szecsey, 2000). Despite the negative results reported in this clinical trial, the anti-inflammatory strategy for AD treatment should not be nullified. The primary end points used in the study might not accurately reflect the contribution of brain inflammation in cognitive decline in AD. The correlation of inflammation and cognitive functions could be examined by testing more biomarkers of inflammation. Also, not seeing the effect of one particular drug prednisone does not nullify the possibility that other drugs may work. In short, hiPSC-derived microglia-like cells can be utilized to interrogate genotype-phenotype relationships in AD, and allow for studying human microglial functions in AD pathology and therapy.

### MS

MS is the most common chronic neurological disorder among children and young adults, which is characterized by inflammation, demyelination, progressive axonal loss, and retrograde neuronal degeneration (Bjartmar and Trapp, 2001; Tallantyre et al., 2010). The disease eventually leads to motor function defects and cognitive impairments. However, the pathogenesis of MS is still unclear. Autoimmune responses, and genetic and environmental factors are believed to be involved in MS (Ascherio and Munger, 2007a,b; Oksenberg and Baranzini, 2010; Hauser et al., 2013). In terms of treatment, the major strategy is anti-inflammatory and immunosuppressive regimens. However, these therapies not only cause a range of side effects but also fail to stop the continuously progressing pathology of MS. Therefore, how to achieve neuroprotection and remyelination becomes the key for MS therapy. In the demyelinated lesions, there seems to exist premyelinating OPCs, astrocytes, microglia, and NPCs (Lucchinetti et al., 2000; Chang et al., 2002; Nicaise et al., 2017). During the remission phase of MS, OPCs can repair the myelin damage to some extent; however, in the progressive phase, endogenous OPCs fail to sufficiently rescue the demyelinated axons (Kuhlmann et al., 2008; Boyd et al., 2013; Thiruvalluvan et al., 2016). Given the remyelination defect of endogenous OPCs in MS, hiPSC-derived OPCs hold promise as donor cells in cell replacement therapies.

hiPSC-derived OPCs can differentiate into OLs and remyelinate damaged axons in MS models (Wang et al., 2013; Douvaras et al., 2014; Thiruvalluvan et al., 2016). Most earlier studies used shiverer mice or cuprizone-induced demyelination mice as MS models. However, there are significant differences between these models and MS patients in terms of clinical and pathological presentations. The later established experimental autoimmune encephalomyelitis (EAE) models appear better for MS modeling. Particularly, non-human primate EAE models so far remain the most closely resembling model for studying the pathogenesis and treatment of MS (Jagessar et al., 2010). Thiruvalluvan et al. reported, for the first time, transplantation of hiPSC-derived OPCs into marmoset EAE models. After the authors examined the proper functionalities of hiPSCs-OPCs *in vitro* and in mice, the cells were injected into the cerebral cortex of marmoset (Thiruvalluvan et al., 2016). At different time points following engraftment, hiPSCs-OPCs show a capacity of survival, migration and remyelination (Thiruvalluvan et al., 2016). This study provided an important theoretical basis for cell replacement therapy of MS.

Some of the clinical trials for MS treatment were aimed to test the safety and/or efficacy of vitamins, antibodies, and growth factors [Vitamin A, NCT01407211, NCT01225289; Vitamin D, NCT01952483; recombinant human monoclonal antibody 22 (rHIgM22), NCT01803867, NCT02398461; recombinant human insulin-like growth factor (rhIGF-1), NCT00001669]. HIgM22 exists in MS patients' serum, which could promote remyelination (Pirko et al., 2004). Another phase I clinical trial has been initiated to culture OPCs from human patients, with a long-term goal of therapeutic application of these cultured cells for treatment of demyelinating diseases (NCT00283023). Interventional strategies for MS need to consider both the microenvironment of the demyelination sites and the selection of cellular grafts. It may not be surprising that a combinatory approach targeting both the niche of lesioned brain and application of OPCs for replacement is tested in future trials. Small molecules and biomaterial scaffold that could promote the survival, differentiation, and/or function of engrafted OPCs, may also be incorporated into the interventional strategy for a better effect.

## Summary and future perspective

It is increasingly clear that glial cells not only have a passive and supporting role but also make other critical contributions in the CNS—they are indispensable for brain homeostasis, development, and functions, and also participate in a variety of neurological diseases. However, the research on glial cells has been hampered by a constellation of challenges that include difficulties in culturing them *in vitro*, limited accessibility of human neural tissues, altered characteristics by the isolation procedure, and insufficiency of rodent animals in modeling human diseases involving glial cells due to the large distinctions in morphology, function, and cell type distribution in humans vs. rodents. New advances in the field of hiPSC technology allow for generation of glial cells by differentiating hiPSCs into various glial cells in defined media, providing a potential platform to better understand the role of glial cells in CNS development and function. hiPSC-derived glial cells can be employed to model some complex neurological diseases, such as ALS, SCI, AD, MS, facilitating the understanding of glia-related mechanisms underlying disease etiology and development.

A few clinical trials have been initiated in which glial cells derived from fetal tissues or hiPSCs would be applied to treat certain neurological diseases, such as ALS, SCI, and AD. Engraftment of glial cells is aimed at either replacing the damaged glia or nourishing the microenvironment of degenerating cells. Despite the robust potential of iPSC derivatives in disease modeling and treatment, caution needs to be taken when moving to the clinical application stage. A number of issues should be taken into account as follows. (i) Caveats of iPSC reprogramming technology. Mutation accumulation is inevitable during the reprogramming process and the subsequent expansion of iPSC clones. Careful examination is necessary to identify any harmful mutations associated with cancer development or functions of the target cells. With regard to the familial disease patients, the reprogramming technology may need to be combined with gene editing technology for correction of the mutant genes. Yet whole genome deep sequencing would be required to investigate any possible off-target effects. Compared to off-shelf drugs, individualized strategy using iPSC derivatives is costly and requires lengthy preparation and validation of donor cell quality and efficacy, which would further limit the number of eligible patients and applicable diseases/indications. The tumorigenic risk has always been a concern associated with application of iPSC derivatives. Any remaining undifferentiated pluripotent cell would pose serious risk after transplantation. A robust differentiation protocol and rigorous sorting step may provide a solution. An alternative cell source is induced neural stem cells, which could give rise to glial cells yet requires a shorter period of time for the differentiation step. In addition, the adult stem cell nature of induced neural stem cells implicates less tumorigenic risk. Furthermore, due to the rapid advancement of field, regulatory bodies need to come up with more practical and timely policies to guide the application of iPSCs and derivatives. Related policies have been lacking or outdated in some countries. (ii) Patient selection. Each patient is unique and the disease condition would normally be much more complex than the condition formulated in an animal model, in which the number of variables is intentionally minimized. Current knowledge is still insufficient to determine the response of individual patient to certain cellular therapy. A better understanding of the influence of genetic, epigenetic, and environmental components on the treatment effect would be essential to identify a responder patient group and the best disease stage for intervention. To achieve this, it is necessary to optimize each step involved in the cellular therapy, such as standardization of cell differentiation, sorting, characterization, and the dosage and route of cell delivery. (iii) Graft tracing. Knowing where the cells go and what cells they become would be critical for evaluation and improvement of the therapy. However, clinical grade tracing technologies are still very limited. Nanoparticle labeling may be used for glia graft tracing through imaging; but there is still a large room for improvement in this field.

To summarize, along with the rapid progress of stem cell biology and cell replacement therapy in the past years, we may witness, hopefully in the foreseeable future, the application of hiPSC-derived glial cells in the clinic for treatment of neurological diseases. Nevertheless, some crucial issues need to be addressed to improve the safety and efficacy of such therapeutic strategies.

## Author contributions

WZ, QL, CZ, and YD searched the literature and drafted the manuscript. H-LZ and ZC critically revised the manuscript, and all authors approved the final version for submission.

### Conflict of interest statement

The authors declare that the research was conducted in the absence of any commercial or financial relationships that could be construed as a potential conflict of interest.
